# Primary infectious aortic aneurysm: a case series and review of the literature

**DOI:** 10.1590/1677-5449.202102061

**Published:** 2022-07-29

**Authors:** Fernanda Beatriz Araújo de Albuquerque, Matheus Oliveira Feijó, Jacob Hindrik Antunes Smit, Ricardo Bernardo da Silva, Adenauer Marinho de Oliveira Góes

**Affiliations:** 1 Universidade Federal do Pará – UFPA, Belém, PA, Brasil; 2 Centro Universitário do Estado do Pará – CESUPA, Belém, PA, Brasil; 3 Santa Casa de Londrina, Londrina, PR, Brasil

**Keywords:** infectious aneurysm, aortic aneurysm, abdominal aortic aneurysm, ruptured aortic aneurysm

## Abstract

Infectious aneurysms, formerly known as mycotic aneurysms, are rare, most often involve the aorta in young patients, and have a greater tendency to rupture than aneurysms of other etiologies. The most characteristic shape is saccular and the most common etiologic agents are *Staphylococcus sp.* and *Salmonella sp*. There is scant and imprecise information in the literature about correct nomenclature, diagnosis, and treatment. The authors present three cases in which diagnostic and therapeutic procedures were documented. In addition to reporting this case series, the authors also present a review of the subject, outlining pertinent diagnostic and therapeutic strategies.

## INTRODUCTION

Infectious aneurysms, formerly known as mycotic aneurysms, are rare, accounting for 1-3% of all aneurysms.^1^^,^^2^ They most frequently affect the aorta^2^^,^^3^ in young men,^4^ the most characteristic shape is saccular,^5^ and they have a greater tendency to rupture than non-infectious aneurysms.^6^^,^^7^ The most common etiologic agents are *Staphylococcus sp.* and *Salmonella sp*.^6^^-^8 Infectious aneurysms may be associated with injection of illicit drugs,^4^^,^^8^ immunosuppression, or sepsis.^1^^,^^4^ Diagnosis is challenging because of the low prevalence and the nonspecific signs and symptoms.^6^^-^8 Fever, pulsating mass, and abdominal pains are described classically, as are elevated inflammatory markers and positive blood cultures, but are not always present.^3^^,^^9^ Treatment for this type of aneurysm must be initiated rapidly, with antibiotic therapy (ideally guided by cultures) and surgery.^1^^,^^6^^-^9 Few surgeons accumulate experience with their treatment and the literature contains imprecise information on nomenclature, diagnostic criteria, and therapeutic strategies. This article presents a series of three cases and highlights aspects of their clinical presentation, diagnosis, and treatment, in addition to reviewing the literature on the subject to help to standardize the management of this uncommon, but extremely serious, disease. This case series has been analyzed and approved by the Ethics Committee at the originating institution, under decision number 48950921.5.0000.5169.

## CASE DESCRIPTIONS

### Case 1

**Figure 1:**
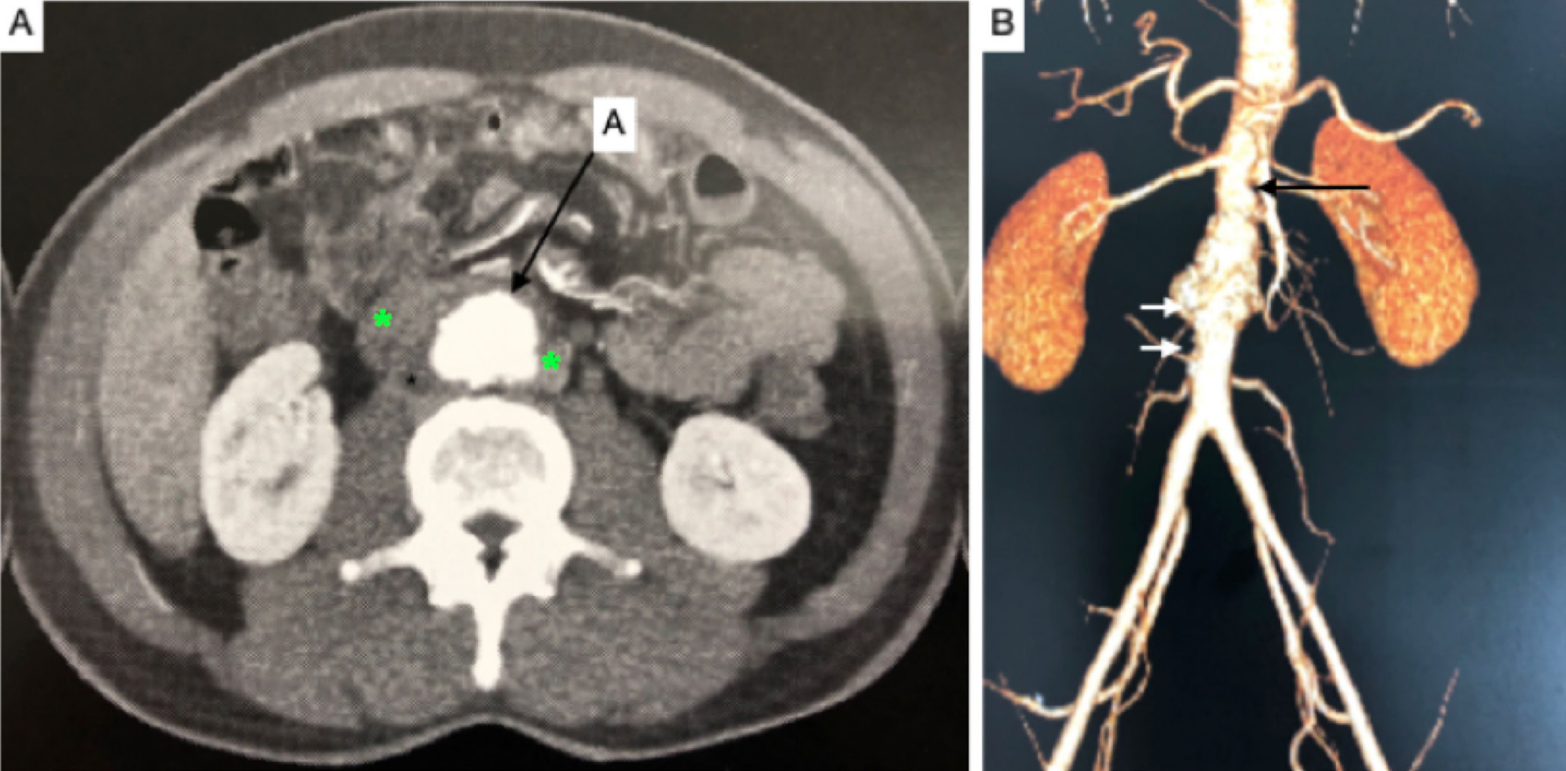
Computed tomography with intravenous contrast. **(A)** Axial slice; A: aorta; the asterisks mark an image compatible with periaortic collection/mass. **(B)** Reconstruction with the maximum intensity projection (MIP) technique. Observe the irregular outlines not just of the aneurysm, but also of the aortic segments proximal (black arrow) and distal (white arrows) of the aneurysm.

**Figure 2:**
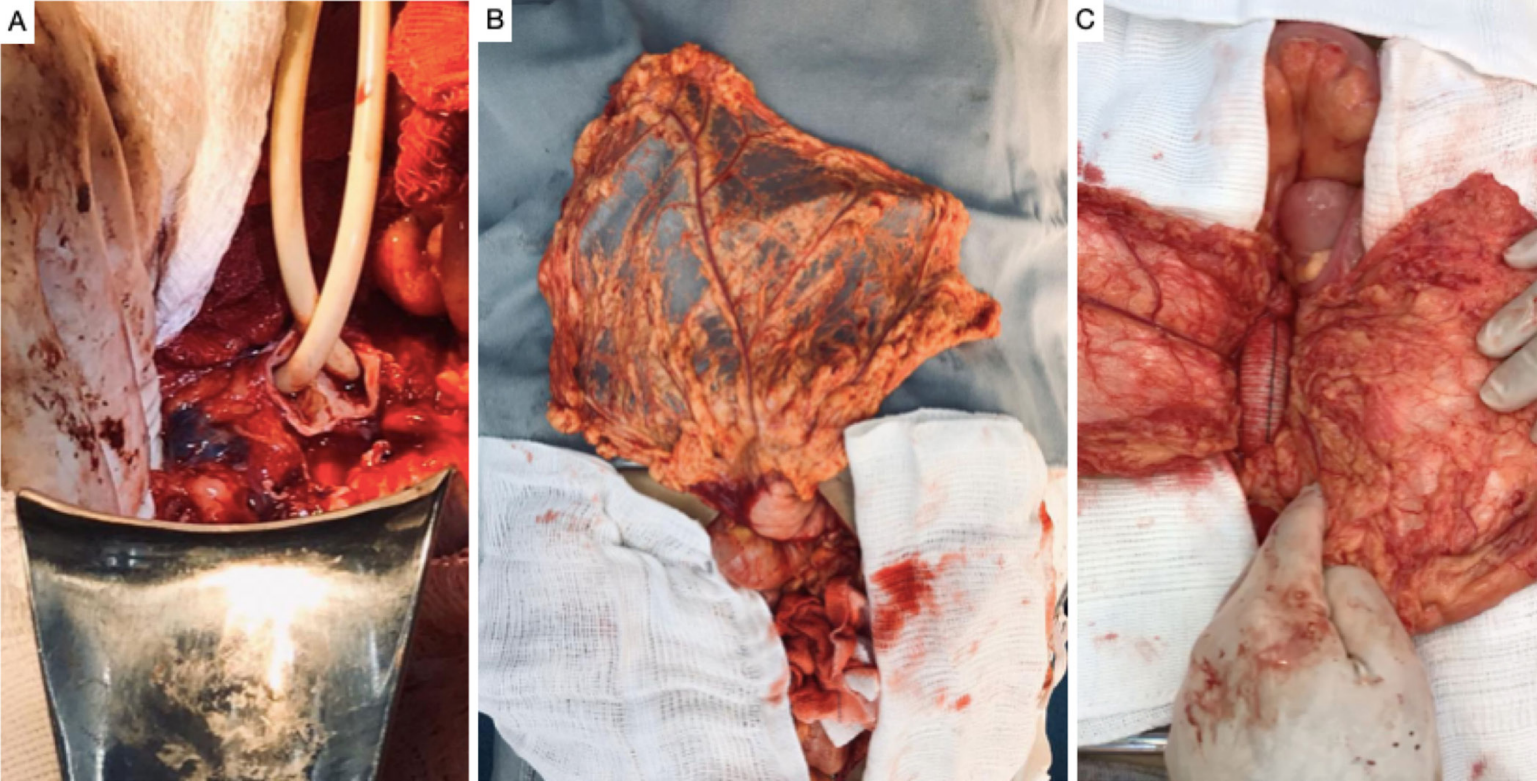
Intraoperative images. **(A)** Temporary hemostasis with endoluminal inflation of Foley catheters in the iliac arteries; **(B)** Vascularized pedicle of the great omentum; **(C)** Pedicle of the great omentum in position to be wrapped around the tubular Dacron graft.

**Figure 3:**
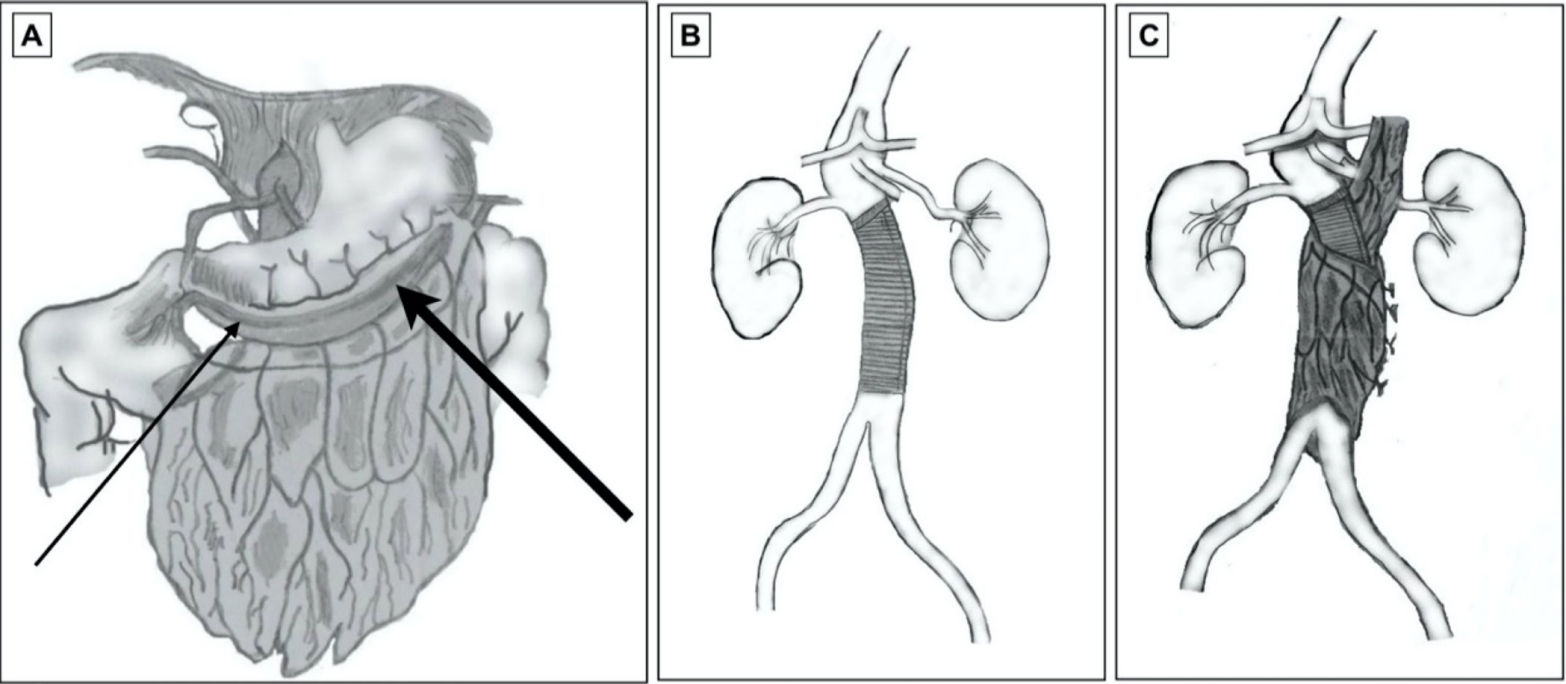
Diagrams illustrating the tubular Dacron graft. **(A)** Omentum, right gastroepiploic artery (thin arrow) and left gastroepiploic artery (thick arrow); **(B)** Abdominal aorta after implantation of the Dacron graft. **C)** Omentum wrapped around the abdominal aortic graft.

A 44-year-old man was referred for follow-up after deep venous thrombosis (DVT). He presented with pain, erythema, and edema involving both legs. He had been treated for erysipelas with penicillin G benzathine and 4 months later suffered a similar episode, with more severe symptoms, restricted to the left lower limb, including edema and clubbing. He was prescribed another course of penicillin and a Doppler ultrasonography examination confirmed DVT of the common, superficial, and deep femoral veins, for which he was prescribed rivaroxaban for 6 months. This treatment had been concluded by the time the patient was seen by the vascular surgeon. However, he still complained of abdominal pains. During the second episode of erysipelas (8 months before the reference consultation), he reported an episode of acute and intense lumbar and abdominal pain, which improved after a few days, persisting at a lower intensity. This pain was attributed to ankylosing spondylitis (diagnosed 13 years previously), for which the patient was using subcutaneous adalimumab injections. He denied fever, weight loss, and using illicit drugs. Physical examination identified a painful pulsating mass with an audible murmur in the mesogastrium. Hemoglobin and leukocytes were within normal limits, but erythrocyte sedimentation rate (ESR) and C-reactive protein (CRP) were elevated (22 and 12, respectively). Angiotomography showed a fusiform aneurysm of the infrarenal aorta, with a maximum diameter of 3.6 cm and lobed outlines, surrounded by hypodense hematoma, with no signs of active bleeding, but with interrupted parietal calcifications and penetrating ulcerations, in addition to an inaccurate definition of the posterior aortic outline, suggestive of an infrarenal abdominal aortic aneurysm with signs of contained rupture (Figure 1). The patient was admitted. Blood cultures were negative and an echocardiogram showed no signs of endocarditis. Intravenous ciprofloxacin and clindamycin were administered for 14 days. During laparotomy on the 15th day, there was no fetid odor or liquid collections, but there was significant fibrosis, thickening of the artery wall, and adherence to adjacent tissues. Proximal control was achieved by infrarenal clamping. Dissection of the distal neck was not feasible because of fibrosis. Distal control was obtained by intraluminal inflation of Foley catheters in the common iliac arteries (Figure 2A). After longitudinal arteriotomy and removal of thrombi, contained rupture of the posterior aneurysm wall was confirmed. The aorta was resected to the maximum extent possible, including its posterior wall, and the stumps of the lumbar arteries were sutured. An aorto-aortic Dacron graft was used for reconstruction, wrapped in a vascularized pedicle of the greater omentum (Figures 2B, 2C, and 3). Cultures of the thrombus and aorta wall were negative. The intravenous antibiotic therapy was maintained up to discharge on the 14th postoperative (PO) day. Oral ciprofloxacin and clindamycin were prescribed for a further 6 weeks and the adalimumab was withdrawn. The patient has been in outpatients follow-up for 6 months.

### Case 2

**Figure 4:**
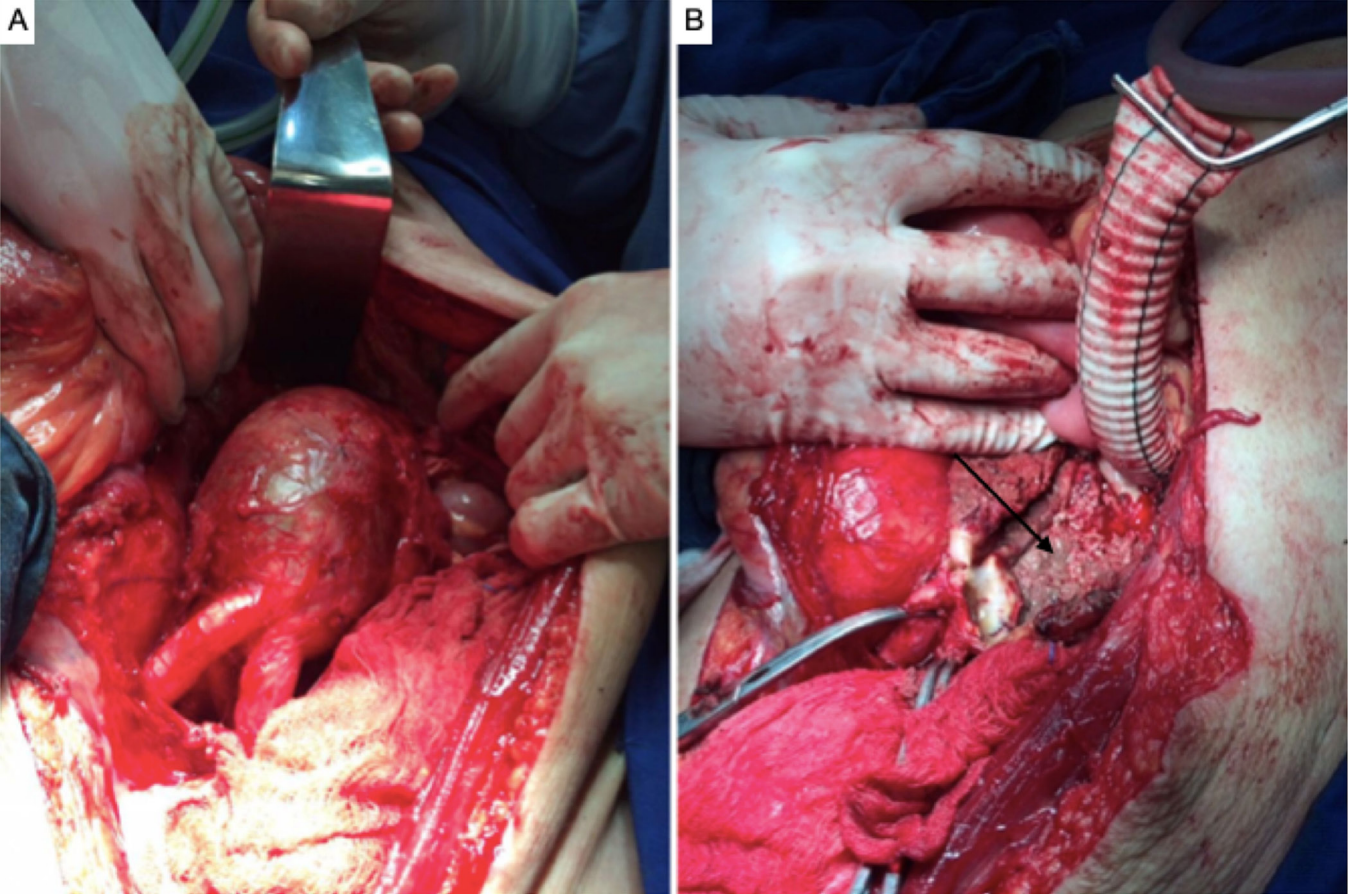
Intraoperative images. **(A)** Large aneurysm of the infrarenal aorta. **(B)** Tubular Dacron graft. The arrow indicates where the posterior wall of the aorta was resected. The proximal anastomosis is already concluded.

A 62-year-old man was admitted for recurrent abdominal pains after outpatient consultations. He reported severe gastroenteritis, treated with antibiotics 4 months previously. He had diffuse abdominal pain, more intense in the mesogastrium; angiotomography confirmed a juxtarenal aortic aneurysm with a diameter of 5.3 cm and signs of periaortic inflammation. He had elevated CRP (34 mg/L) and leukocytosis at 17,000/µL. Blood cultures were negative. After 7 days on intravenous ciprofloxacin and clindamycin, the patient was operated. The following interoperative findings were observed: intestinal loops with signs of inflammation and a fetid odor after the aneurysm sac was opened. Proximal supraceliac clamping was performed prior to reconstruction with an 18 mm Dacron aorto-aortic graft (Figure 4). During the postoperative period, the patient developed renal dysfunction and nosocomial pneumonia; an aorta wall culture revealed *Escherichia coli*, and the antibiotic therapy was changed to piperacillin with tazobactam and vancomycin. The patient died on the ninth PO day.

### Case 3

**Figure 5:**
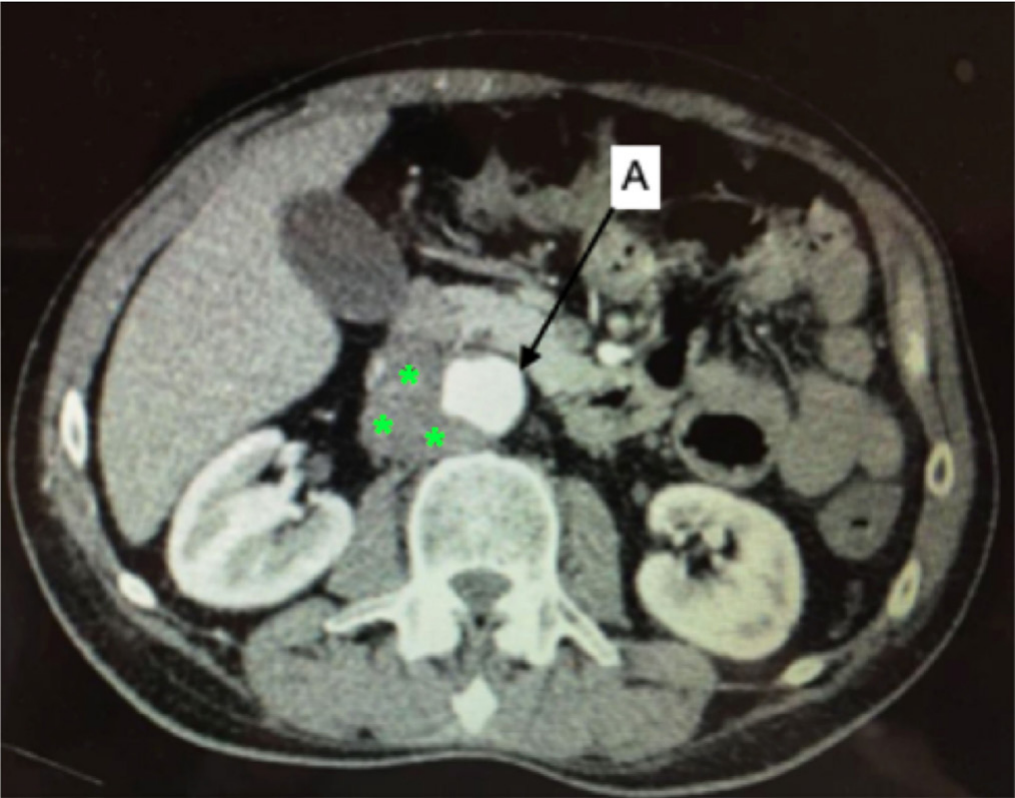
Computed tomography with intravenous contrast, axial slice. **(A)** Aorta; the asterisks mark an image compatible with periaortic collection/mass.

**Figure 6:**
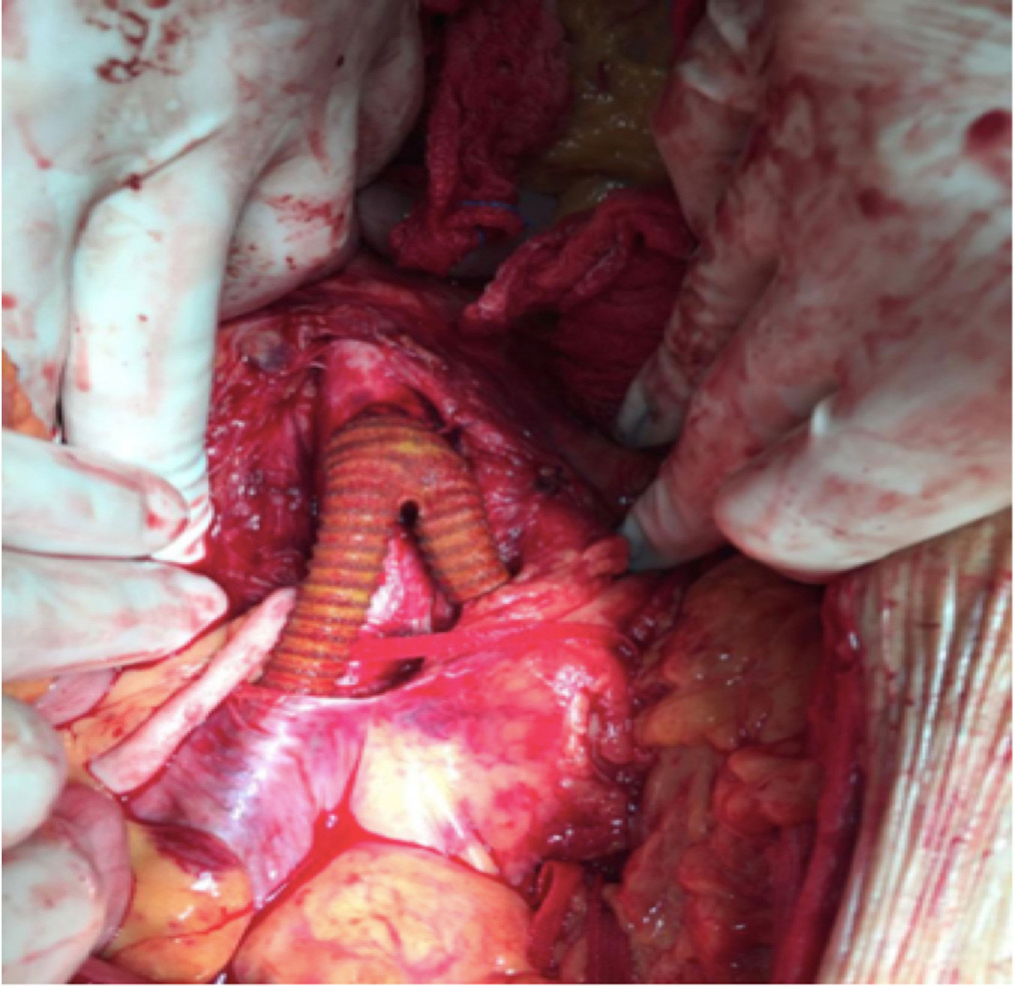
Intraoperative images. Aortoiliac graft with bifurcated silver-impregnated Dacron prosthesis.

A 59-year-old man with a prior history of admissions for psychiatric conditions was admitted because of abdominal pains. On physical examination, he reported pain in response to deep palpation of the mesogastrium, with a pulsating mass. Angiotomography confirmed a 5.7 cm juxtarenal saccular aneurysm and periaortic collection (Figure 5). His ESR was normal, CRP was elevated (27 mg/L), and he had leukocytosis (21,000/µL). Blood cultures were positive for coagulase-negative *Staphylococcus* and antibiotic therapy was started with teicoplanin and piperacillin with tazobactam. After 7 days, by when blood cultures were already negative, the patient underwent surgical treatment. Supraceliac clamping was needed to achieve proximal control of the aorta. When the aneurysm sac was opened, an intense odor was noted and a contained rupture of the posterior wall was observed. Reconstruction was performed with an 18x9 mm bifurcated Dacron aortoiliac graft wrapped in a vascularized pedicle of the greater omentum (Figure 6). Antibiotic therapy was maintained for 30 days postoperatively. One year after discharge, the patient underwent surgery for bilateral degenerative aneurysms of the common femoral artery (infectious etiology was ruled out). The patient is in outpatients follow-up 2 years after treatment of the infected aortic aneurysm.

One feature that the three cases described in this series all have in common is that angiotomography did not show periaortic gas.

## DISCUSSION

The term mycotic aneurysm was coined by William Osler in 1885,^10^ because of the mushroom-like appearance of the aneurysmal lesions.^10^^,^^11^ However, the term induces the erroneous idea that etiology is fungal.^5^^,^^11^ The name infectious aortitis could denote several different conditions, such as aortoenteric fistulas and infections after surgical manipulation, including infections of aortic grafts.^5^^,^^8^^,^^12^^-^14 The best term is primary infectious aneurysm, which denotes a dilation secondary to infection of the aorta wall.^2^^,^^5^^,^^11^^,^^14^^,^^15^ The condition is rare,^1^^,^^5^^-^7^,^^9^^,^^16^ but highly lethal.^6^^-^7^,^^9^^,^^16^

In the West, infectious aneurysms account for no more than 3% of all aneurysms^1^^,^^2^^,^^5^^,^^6^^,^^8^^,^^9^^,^^11^^,^^14^^,^^16^^-^18 and tend to occur in men^8^^,^^14^ who are younger than those who develop degenerative aneurysms.^4^^,^^7^^,^^11^ These aneurysms can grow rapidly and there is a high risk of rupture,^2^^,^^4^^,^^8^^,^^9^^,^^12^^,^^16^^,^^17^ with mortality rates as high as 60%.^2^ The following etiologic agents have been reported: gram-positive bacteria such as *Staphylococcus sp.*,^2^^,^^4^^,^^6^^-^8^,^^11^^,^^14^^,^^19^*Enterococcus sp.*,^11^*Streptococcus sp.*^2^^,^^8^^,^^11^^,^^18^^,^^19^ and *Clostridium sp.*,^11^ and gram-negative bacteria such as *Salmonella sp.*,^2^^-^4^,^^6^^-^8^,^^11^^,^^14^*Pasteurella sp.*,^7^*Brucella sp.*,^20^*Coxiella burnetti,*^11^ and *Pseudomonas aeruginosa*,^2^^,^^19^ in addition to fungi.^5^^,^^11^^,^^18^ The most frequently identified agents are members of the *Staphylococcus* and *Salmonella* genera.^6^^,^^12^^,^^18^ The source of infection is not identified in 1/3 of cases and the etiologic agent is not established in 20-40% of cases.^11^^,^^14^^,^^15^ Infectious aneurysms can be caused by contiguity^21^ or, frequently, by bacteremia.^1^^,^^2^^,^^5^^,^^11^^,^^14^^,^^17^^,^^19^ After attaching to the artery wall, the microorganism provokes acute inflammation with neutrophilic infiltration, leading to activation of enzymes and weakening of the artery wall,^8^ resulting in suppuration and arterial dilatation.^1^^,^^2^^,^^5^^,^^9^^,^^11^^,^^17^^,^^20^^,^^22^ Aortic involvement is more common because of the more pronounced vasa vasorum of larger caliber arteries, which facilitates bacterial colonization.^8^^,^^11^^,^^14^^,^^19^

Early diagnosis is key to therapeutic success.^6^^,^^8^^,^^9^^,^^12^^,^^16^ Classically, there is fever,^1^^,^^3^^,^^4^^,^^6^^-^8^,^^14^^,^^18^ abdominal/lumbar pains, and a pulsating mass^1^^,^^4^^,^^6^^-^8^,^^14^^,^^18^ in the presence of an infectious condition (osteomyelitis, urinary infections, tuberculosis, gastroenteritis, and soft tissue infections)^1^^,^^4^^,^^6^^,^^11^^,^^14^ and/or immunosuppression caused by diseases or medications (cancer, renal failure requiring dialysis, HIV, diabetes, corticosteroids). However, asymptomatic cases can also occur.^4^^,^^14^ All three of the cases reported above had abdominal pains; cases 1 and 2 had a history of an infectious condition, and there was also use of immunosuppressant medication in case 1. Laboratory tests generally show Leukocytosis^1^^,^^6^^,^^8^^,^^11^^,^^12^^,^^14^ and inflammatory markers such as elevated ESR and CRP,^6^^,^^12^ in addition to positive blood cultures.^1^^,^^3^^,^^4^^,^^6^^,^^7^^,^^16^ However, blood cultures can be negative even during the acute phase in up to 50% of cases,^14^^,^^23^ particularly in patients who are being given antibiotics, which is common.^8^^,^^14^^,^^15^ Two of the cases in the present series had positive blood cultures. Angiotomography will often show parietal irregularities, saccular dilatations, changes suggestive of inflammation, perivascular liquid collections/masses, periaortic gas buildup, signs of free or contained rupture, and rapid progression over a series of examinations.^1^^,^^4^^,^^5^^,^^8^^,^^9^^,^^11^^,^^12^^,^^14^^,^^17^^,^^24^ Hepatic and splenic abscesses should be sought on tomography.^9^^,^^21^ Echocardiography is a convenient method for investigating endocarditis.^4^ Positron emission tomography/computed tomography scanning (PET-CT SCAN) is a tool with high diagnostic accuracy and very good sensitivity, but its specificity is affected by false-positive results in cases of inflammatory aneurysms and arterites.^24^

There is no consensus on how to define the primary infectious etiology of these aneurysms.^5^^,^^15^^,^^17^ It is suggested that diagnosis is based on the combination of clinical status, laboratory tests, and tomographic findings^1^^,^^6^^,^^12^^,^^17^^,^^21^ (Table 1). It is essential to be clear that this diagnosis can be made in the absence of fever and positive blood cultures.^1^^,^^3^^,^^8^^,^^9^^,^^15^^,^^16^^,^^23^ After drawing blood for cultures, antibiotic therapy for *Staphylococcus sp.* (vancomycin) and antibiotic therapy should be initiated for *Salmonella sp.* (quinolones or third-generation cephalosporins),^1^^,^^4^^,^^11^^,^^12^^,^^15^^,^^16^^,^^23^ to be changed or supplemented with antifungals, depending on the results. There is no consensus on the duration of treatment.^6^^-^8^,^^11^^,^^19^ The majority of authors suggest preoperative intravenous antibiotic therapy for 1 to 6 weeks.^4^^,^^6^^,^^12^ Clinical treatment alone, with antibiotics, is associated with mortality exceeding 80%.^13^^,^^14^^,^^23^

**Table 1:** Criteria suggestive of infectious aneurysm etiology.

**Clinical presentation**	Abdominal/lumbar pains
	Fever
	Sepsis/shock
**Laboratory**	Elevated C-reactive protein
	Elevated leukocytes
	Positive blood/aortic tissue culture
**Computed tomography**	Saccular/multilobed outlines
	Periaortic gas
	Amorphous periaortic mass/collection
	Rapid expansion (days)
	Rupture
	Location in an atypical aortic segment (for example, paravisceral)
	Multiple aneurysms

Source: compiled by the authors, 2021.

Surgical treatment can be with endovascular procedures or conventional surgery.^1^^,^^3^^,^^6^^,^^7^^,^^9^^,^^14^^,^^15^^,^^21^^,^^23^ Endovascular treatment of infectious aneurysms of the thoracic aorta was described in 1998.^25^ Since then, several other reports have been published.^1^^,^^3^^,^^6^^,^^8^^,^^12^^,^^15^^,^^21^^,^^23^ This strategy is less invasive because it avoids dissections in an anatomy compromised by infection and the aorta is not clamped, which in theory benefits patients with a high surgical risk.^1^^,^^7^^,^^11^ However, implantation of endoprostheses in infected tissues increases the incidence of complications, including endoprosthesis infection,^1^^,^^6^^,^^9^^,^^23^ and also of malpositioning and consequent endoleaks with the potential for aneurysm rupture.^4^^,^^8^ Other undesirable outcomes include sepsis, fistulas,^8^ and expansion of the aneurysm.^16^ It is undeniable that endovascular treatment does not enable the removal of infected tissues^9^^,^^12^^,^^16^^,^^23^ and that there is a high risk of merely delaying open surgery, adding the need for explantation of the endoprosthesis to an operation that is already highly complex.

Conventional surgery is associated with morbidity and mortality of up to 44%^12^ and remains the gold standard^11^^,^^12^^,^^16^^,^^22^ because, although recovery is slower, reintervention rates are lower.^3^^,^^6^ In addition to resection of the aneurysm,^9^^,^^12^^,^^13^ surgery should involve extensive retroperitoneal debridement, circumferential aortic resection,^9^^,^^13^^,^^16^^,^^22^^,^^23^ and *in situ* or extra-anatomic revascularization with prosthetic grafts or allografts.^1^^,^^9^^,^^11^^-^13^,^^16^^,^^22^ Use of an extra-anatomic bypass avoids grafting in an infected field; however, rupture of the stump of the aorta, lower limb amputation, or reinfection can occur.^8^^,^^13^ In turn, with *in situ* revascularization, using a graft impregnated with silver or antibiotics,^13^ anastomosis in the infected bed involves the risk of dehiscence and pseudoaneurysm formation.^1^ There are insufficient data to compare complications associated with *in situ* and extra-anatomic grafts, but they are more common with extra-anatomic grafts.^13^^,^^22^ Studies report mortality of 5-49% for *in situ* grafts vs. 24-50% for extra-anatomic grafts, while infectious complications occur in approximately 20% of cases with both strategies.^11^

Dissection in the midst of thickened/adherent tissues increases the risk of bleeding and iatrogenic injuries, primarily involving the vena cava and ureters. Use of double J stents in advance can help to identify the ureters within the thickened retroperitoneal space,^21^^,^^22^ as was done in case 1. If dissection of the distal neck is not possible, clamping can be substituted by endovascular occlusion of the iliac arteries with Foley catheters,^1^^,^^23^ as was done in cases 1 and 3. The prosthesis can be isolated from adjacent tissues by wrapping it with a vascularized pedicle of the greater omentum^2^^,^^11^^,^^22^^,^^23^ as in cases 1 and 3; vascularized omentum also improves the delivery of antibiotics to the prosthesis.^2^^,^^11^^,^^22^ There are also descriptions of cryopreserved cadaveric aorta (not available in Brazil) in reconstruction using grafts made from both femoral veins.^11^^,^^13^^,^^22^ Technical details that improve the results of surgery include the use of double J stents to identify the ureters, preparation of the colon to reduce the need to displace loops out of the cavity, central venous access, invasive blood pressure monitoring, use of an 8F angiographic introducer in the internal jugular to enable rapid infusion of blood products, fluid balance positive by at least 1,000 mL before the conclusion of the procedure, and heating with a thermal blanket. There is no consensus on the duration of postoperative antibiotic therapy.^8^ Some authors recommend 6 weeks,^6^^,^^15^^,^^18^^,^^22^ while other suggest 3 to 6 months,^4^^,^^6^^,^^7^^,^^11^^,^^12^^,^^15^ or even lifelong antibiotic therapy.^6^^,^^12^^,^^22^

Rare diseases and complex treatment demand sharing of information; discussion with colleagues who are experts in aortic surgery and sharing experiences in groups that practice collective intelligence^26^ are important to increase the likelihood of success. Infectious etiology should always be considered when faced with fever and abdominal/lumbar pains with a pulsating mass, particularly in the presence of a confirmed infection or immunosuppression caused by diseases/medications and if leukocytes and inflammatory markers are elevated. Blood cultures are often negative. Suggestive angiotomographic images include saccular dilatations, perivascular collections, and contained ruptures. Waiting for “classic” presentations and positive blood cultures before initiating the correct treatment can compromise the patient’s prognosis.

For postoperative control, it is recommended that computed tomography angiographies should be conducted at 1 and 6 months and annually thereafter, to check for complications and a need for reintervention.^12^

Limitations of this series include the small number of cases and the lack of documentation of imaging exams conducted for postoperative follow-up of the patients.

## References

[1] Hurtado DFG, Neira JCH, Atala SH, Lawrence PT (2019). Manejo de un aneurisma infeccioso. Rev Cir.

[2] Dsouza R, Kota AA, Jain S, Agarwal S (2020). Mycotic abdominal aortic aneurysm complicated by infective spondylitis due to Pseudomonas aeruginosa. BMJ Case Rep.

[3] Nagrodzki J, Sharrocks KE, Wong VK, Carmichael AJ (2020). A mycotic aneurysm related to Salmonella Rissen infection: a case report. BMC Infect Dis.

[4] Kordzadeh A, Watson J, Panayiotopolous YP (2016). Mycotic aneurysm of the superior and inferior mesenteric artery. J Vasc Surg.

[5] Sörelius K, di Summa PG (2018). On the diagnosis of mycotic aortic aneurysms. Clin Med Insights Cardiol.

[6] Guo Y, Bai Y, Yang C, Wang P, Gu L (2018). Mycotic aneurysm due to Salmonella species: clinical experiences and review of the literature. Braz J Med Biol Res.

[7] Kano Y, Takamatsu A, Honda H (2020). Mycotic aneurysm due to Pasteurella multocida. QJM.

[8] Deipolyi AR, Czaplicki CD, Oklu R (2018). Inflammatory and infectious aortic diseases. Cardiovasc Diagn Ther.

[9] Zeng Z, Li Z, Zhao Y (2020). Endovascular repair combined with staged drainage for the treatment of infectious aortic aneurysm: a case report. BMC Cardiovasc Disord.

[10] Osler W (1885). The gulstonian lectures, on malignant endocarditis. Br Med J.

[11] Wanhainen A, Verzini F, Van Herzeele I (2019). Editor’s Choice - European Society for Vascular Surgery (ESVS) 2019 Clinical Practice Guidelines on the Management of Abdominal Aorto-iliac Artery Aneurysms. Eur J Vasc Endovasc Surg.

[12] Zhu C, Zhao J, Huang B, Yuan D, Yang Y, Wang T (2020). Long‐term outcome of endovascular aortic repair for mycotic abdominal aortic aneurysm. ANZ J Surg.

[13] Berchiolli R, Mocellin DM, Marconi M (2019). Ruptured mycotic aneurysm after intravesical instillation for bladder tumor. Ann Vasc Surg.

[14] Wilson WR, Bower TC, Creager MA (2016). Vascular Graft Infections, Mycotic Aneurysms, and Endovascular Infections: a scientific statement from the American Heart Association. Circulation.

[15] Dang Q, Van Eps RG, Wever JJ (2020). Nationwide study of the treatment of mycotic abdominal aortic aneurysms comparing open and endovascular repair in The Netherlands. J Vasc Surg.

[16] Sörelius K, Wanhainen A, Furebring M (2016). Nationwide study of the treatment of mycotic abdominal aortic aneurysms comparing open and endovascular repair. Circulation.

[17] Wang TKM, Griffin B, Cremer P (2020). Diagnostic utility of CT and MRI for Mycotic Aneurysms: a meta-analysis. AJR Am J Roentgenol.

[18] Watanabe N, Koyama S, Tabira M (2021). Infected aortic aneurysm caused by Streptococcus pyogenes: a case report. J Infect Chemother.

[19] Matsuo T, Mori N, Mizuno A (2020). Infected aortic aneurysm caused by Helicobacter cinaedi: case series and systematic review of the literature. BMC Infect Dis.

[20] Alhaizaey A, Alassiri M, Alghamdi M, Alsharani M (2016). Mycotic aortic aneurysm due to brucellosis. J Vasc Surg Cases Innov Tech..

[21] Patel AP, Cantos A, Butani D (2020). Mycotic Aneurysm of the Hepatic Artery: a case report and its management. J Clin Imaging Sci.

[22] Tshomba Y, Sica S, Minelli F (2020). Management of mycotic aorto-iliac aneurysms: a 30-year monocentric experience. Eur Rev Med Pharmacol Sci.

[23] Kazuno K, Kinoshita H, Hori M (2020). Endovascular treatment for mycotic aneurysm using pyoktanin- applied devices. Cvir Endovascular..

[24] Husmann L, Huellner MW, Ledergerber B (2020). Diagnostic accuracy of PET/CT and contrast enhanced CT in patients with suspected infected aortic aneurysms. Eur J Vasc Endovasc Surg.

[25] Semba CP, Sakai T, Slonim SM (1998). Mycotic aneurysms of the thoracic aorta: repair with use of endovascular stent-grafts. J Vasc Interv Radiol.

[26] Erzinger FL, de Araujo WJB, Ordinola AAM (2018). Vascular Forum: collective intelligence in the resolution of vascular clinical cases. J Vasc Bras.

