# Supplementary material for: Primary infectious aortic aneurysm: a case series and review of the literature
Source: J Vasc Bras. 2022 Jul 29;21:e20210206. doi: 10.1590/1677-5449.202102061 (PMC9351984; doi:10.1590/1677-5449.202102061)
Supplement: Translation

# Translation: Aneurisma infeccioso primário da aorta: série de casos e revisão da literatura

**DOI:** 10.1590/1677-5449.202102062

**Authors:** Fernanda Beatriz Araújo de Albuquerque, Matheus Oliveira Feijó, Jacob Hindrik Antunes Smit, Ricardo Bernardo da Silva, Adenauer Marinho de Oliveira Góes Junior

**Affiliations:** 1 Universidade Federal do Pará – UFPA, Belém, PA, Brasil; 2 Centro Universitário do Estado do Pará – CESUPA, Belém, PA, Brasil; 3 Santa Casa de Londrina, Londrina, PR, Brasil

**Keywords:** aneurisma infectado, aneurisma aórtico, aneurisma da aorta abdominal, aneurisma aórtico roto

## Abstract

Aneurismas infecciosos, anteriormente chamados de aneurismas micóticos, são raros; acometem com maior frequência a aorta de pacientes jovens e apresentam maior tendência à rotura do que aneurismas de outras etiologias. O formato sacular é o mais característico, e os agentes etiológicos mais comuns são *Staphylococcus sp* e *Salmonella sp*. A literatura fornece informações limitadas e imprecisas sobre a correta nomenclatura, diagnóstico e tratamento da doença. Os autores reuniram três casos cujos procedimentos diagnósticos e terapêuticos foram documentados. Além de relatar essa série de casos, realiza-se uma revisão sobre o tema, a fim de estabelecer estratégias diagnósticas e terapêuticas pertinentes.

## INTRODUÇÃO

Aneurismas infecciosos, anteriormente chamados de aneurismas micóticos, são raros: correspondem a 1-3% entre todos os aneurismas^1^^,^^2^. Com maior frequência acometem a aorta^2^^,^^3^ de homens jovens^4^; o formato sacular é o mais característico^5^ e há maior tendência à rotura do que em aneurismas não infecciosos^6^^,^^7^. Os agentes etiológicos mais comuns são *Staphylococcus sp* e *Salmonella sp*^6^^-^8. Aneurismas infecciosos podem estar associados a uso de drogas ilícitas injetáveis^4^^,^^8^, imunossupressão ou sepse^1^^,^^4^. O diagnóstico é dificultado pela baixa prevalência e pela inespecificidade de sinais e sintomas^6^^-^8. Febre, massa pulsátil e dor abdominal são classicamente descritas, assim como marcadores inflamatórios elevados e hemoculturas positivas; porém, isso nem sempre acontece^3^^,^^9^. Esse tipo de aneurisma requer rápida instituição do tratamento, que engloba antibioticoterapia (idealmente orientada por culturas) e cirurgia^1^^,^^6^^-^9. Poucos cirurgiões acumulam experiência no tratamento, e a literatura fornece informações imprecisas sobre nomenclatura, critérios diagnósticos e estratégias terapêuticas. Este artigo apresenta uma série de três casos e salienta aspectos de apresentação clínica, diagnóstico e terapêutica, além de revisar a literatura sobre o tema para ajudar a padronizar a abordagem dessa doença incomum, porém extremamente grave. Esta série de casos foi apreciada e aprovada pelo Comitê de Ética da instituição de origem, sob o parecer de número 48950921.5.0000.5169.

## DESCRIÇÃO DOS CASOS

### Caso 1

Homem, 44 anos, encaminhado para acompanhamento após trombose venosa profunda (TVP). Apresentava dor, eritema e edema em ambas as pernas. Havia feito tratamento de erisipela com penicilina G benzatina e, após 4 meses, apresentou novo quadro semelhante, com sintomas mais exuberantes e restritos ao membro inferior esquerdo, acompanhados de edema e empastamento. Fez-se novo ciclo de penicilina, e exame de eco-Doppler confirmou TVP de veias femorais comum, superficial e profunda, para a qual foi prescrita rivaroxabana por 6 meses. Quando o paciente foi avaliado pelo cirurgião vascular, o tratamento já havia sido concluído. No entanto, referia dor abdominal. Durante o segundo episódio de erisipela (8 meses antes da consulta), relatou episódio de dor lombar e abdominal aguda e intensa, que melhorou após alguns dias, persistindo com menor intensidade. A dor foi atribuída à espondilite anquilosante (diagnosticada há 13 anos), para a qual o paciente usava injeções subcutâneas de adalimumabe. Negava febre, perda de peso e uso de drogas ilícitas. Ao exame físico, apresentava massa pulsátil dolorosa com sopro audível no mesogástrio. Hemoglobina e leucócitos estavam dentro dos valores de referência; porém, velocidade de hemossedimentação (VHS) e proteína C reativa (PCR) encontravam-se elevadas (22 e 12, respectivamente). A angiotomografia mostrou aneurisma fusiforme da aorta infrarrenal, com diâmetro máximo de 3,6 cm e contornos lobulados, circundado por hematoma hipodenso, sem sinais de sangramento ativo, porém com descontinuidade das calcificações parietais e ulcerações penetrantes, além de perda da definição do contorno aórtico posterior, o que sugeria aneurisma de aorta abdominal infrarenal com sinais de rotura contida (Figura 1). O paciente foi internado. Hemoculturas foram negativas e ecocardiograma não apresentou sinais de endocardite. Administraram-se ciprofloxacina e clindamicina endovenosas por 14 dias. À laparotomia, no 15º dia, não foram observados odor fétido ou coleção líquida, porém havia intensa fibrose, espessamento da parede arterial e aderência dos tecidos adjacentes. O controle proximal foi obtido por clampeamento infrarrenal. Não foi possível a dissecção do colo distal devido à fibrose. O controle distal foi estabelecido pela insuflação intraluminal de sondas de Foley nas ilíacas comuns (Figura 2A). Após arteriotomia longitudinal e retirada de trombos, confirmou-se rotura contida na parede posterior do aneurisma. A aorta foi ressecada o máximo possível, inclusive sua parede posterior, com sutura dos cotos das artérias lombares. Para reconstrução, utilizou-se enxerto aorto-aórtico com prótese de Dacron de 20 mm de diâmetro, envolvido por um pedículo vascularizado do grande omento (Figuras 2B, 2C e 3). Culturas de trombo e parede aórtica foram negativas. A antibioticoterapia endovenosa foi mantida até a alta, no 14º dia de pós-operatório (PO). Foram prescritas ciprofloxacina e clindamicina por via oral por mais 6 semanas, e o uso de adalimumabe foi suspenso. O paciente está em acompanhamento ambulatorial há 6 meses.

**Figura 1:** Tomografia computadorizada com contraste endovenoso. **(A)** Corte axial; A: aorta; os asteriscos correspondem a imagem compatível com coleção/massa periaórtica; **(B)** Reconstrução com técnica *maximum intensity projection* (MIP). Observar as irregularidades de contorno não apenas do aneurisma mas também dos segmentos aórticos proximais (seta preta) e distais (setas brancas) ao aneurisma.

**Figura 2:** Intraoperatório. **(A)** Hemostasia temporária com insuflação endoluminal de sondas de Foley nas artérias ilíacas; **(B)** Pedículo vascularizado do grande omento; **(C)** Pedículo do grande omento já posicionado para envolver o enxerto tubular de Dácron.

**Figura 3:** Representação de enxerto tubular de Dácron. **(A)** Omento, artéria gastromental direita (seta fina) e artéria gastromental esquerda (seta grossa); **(B)** Aorta abdominal após enxerto de Dácron; (**C)** Omento envolvendo a prótese na aorta abdominal.

### Caso 2

Homem, 62 anos, internado por dor abdominal recorrente após atendimentos ambulatoriais. Relatava gastroenterite grave, tratada com antibioticoterapia 4 meses antes. Havia dor abdominal difusa, mais intensa no mesogástrio; angiotomografia confirmou aneurisma aórtico justarrenal de 5,3 cm de diâmetro, com sinais inflamatórios periaórticos. O paciente apresentava PCR elevado (34 mg/L) e leucocitose de 17.000/µL; hemocultura foi negativa. Após 7 dias de ciprofloxacina e clindamicina endovenosas, o paciente foi operado. Observaram-se os seguintes achados operatórios: alças intestinais com aspecto inflamatório e odor fétido após abertura do saco aneurismático. Foram realizados clampeamento proximal supracelíaco e reconstrução com enxerto aorto-aórtico com prótese de Dacron de 18 mm (Figura 4). No pós-operatório, o paciente evoluiu com disfunção renal e pneumonia hospitalar; a cultura da parede aórtica evidenciou *Escherichia coli*, e a antibioticoterapia foi modificada para piperacilina com tazobactam e vancomicina. O paciente foi a óbito no 9º PO.

**Figura 4:** Intraoperatório. **(A)** Volumoso aneurisma de aorta infrarrenal; **(B)** Enxerto tubular de Dácron. A seta aponta a área onde a parede posterior da aorta foi ressecada, anastomose proximal já concluída.

### Caso 3

Homem, 59 anos, com internações anteriores por quadros psiquiátricos, admitido por dor abdominal. Ao exame físico, apresentava dor à palpação profunda do mesogástrio com massa pulsátil. A angiotomografia confirmou aneurisma justarrenal sacular de 5,7 cm e coleção periaórtica (Figura 5). O paciente apresentava VHS normal, PCR elevada (27 mg/L) e leucocitose (21.000/µL). Hemocultura demonstrou *Staphylococcus* coagulase negativo, e iniciou-se antibioticoterapia com teicoplanina e piperacilina com tazobactam. Após 7 dias, já com a hemocultura negativa, foi realizado tratamento cirúrgico. O controle aórtico proximal requereu clampeamento supracelíaco. Odor forte e rotura contida de parede posterior foram notados à abertura do saco aneurismático. A reconstrução foi com enxerto aorto-ilíaco com prótese bifurcada de Dacron 18x9 mm envolvido com pedículo vascularizado do grande omento (Figura 6). No PO, a antibioticoterapia foi mantida por 30 dias. Um ano após a alta, o paciente foi submetido a cirurgia por aneurismas degenerativos bilaterais de artéria femoral comum (etiologia infecciosa descartada). Encontra-se em seguimento ambulatorial 2 anos após tratamento do aneurisma aórtico infeccioso.

**Figura 5:** Tomografia computadorizada com contraste endovenoso, corte axial. **(A)** Aorta; os asteriscos correspondem a imagem compatível com coleção/massa periaórtica.

**Figura 6:** Intraoperatório. Enxerto aorto-ilíaco com prótese bifurcada de Dácron impregnada com prata.

Um ponto comum aos três casos descritos nesta série é o fato da angiotomografia não ter demonstrado gás periaórtico.

## DISCUSSÃO

O termo aneurisma micótico foi cunhado por William Osler em 1885^10^, devido ao aspecto de cogumelo das lesões aneurismáticas^10^^,^^11^. Porém, induzia à ideia equivocada de etiologia fúngica^5^^,^^11^. A denominação aortite infecciosa pode corresponder a várias situações, como fístulas aortoentéricas e infecções após manipulação cirúrgica, inclusive infecções de enxertos aórticos^5^^,^^8^^,^^12^^-^14. A melhor denominação é aneurisma infeccioso primário, que representa dilatação secundária à infecção parietal da aorta^2^^,^^5^^,^^11^^,^^14^^,^^15^. É raro^1^^,^^5^^-^7^,^^9^^,^^16^, porém altamente letal^6^^-^7^,^^9^^,^^16^.

No Ocidente, aneurismas infecciosos não ultrapassam 3% entre todos os aneurismas^1^^,^^2^^,^^5^^,^^6^^,^^8^^,^^9^^,^^11^^,^^14^^,^^16^^-^18 e tendem a acometer homens^8^^,^^14^ mais jovens do que aqueles com aneurismas degenerativos^4^^,^^7^^,^^11^. O crescimento pode ser rápido, com alto risco de ruptura^2^^,^^4^^,^^8^^,^^9^^,^^12^^,^^16^^,^^17^ e mortalidade atingindo 60%^2^. Como agentes etiológicos, já foram descritas bactérias gram-positivas, como *Staphylococcus sp*^2^^,^^4^^,^^6^^-^8^,^^11^^,^^14^^,^^19^, *Enterococcus sp*^11^, *Streptococcus sp*^2^^,^^8^^,^^11^^,^^18^^,^^19^ e *Clostridium sp*^11^, e gram-negativas, como *Salmonella sp*^2^^-^4^,^^6^^-^8^,^^11^^,^^14^, *Pasteurella sp*^7^, *Brucella sp*^20^, *Coxiella burnetti*^11^ e *Pseudomonas aeruginosa*^2^^,^^19^, além de fungos^5^^,^^11^^,^^18^. Os agentes mais frequentemente identificados pertencem aos gêneros *Staphylococcus sp* e *Salmonella sp*^6^^,^^12^^,^^18^. A fonte da infecção não é localizada em 1/3 dos casos, e o agente etiológico não é estabelecido em 20-40% dos casos^11^^,^^14^^,^^15^. Aneurismas infecciosos ocorrem por contiguidade^21^ ou, frequentemente, por bacteremia^1^^,^^2^^,^^5^^,^^11^^,^^14^^,^^17^^,^^19^. Após se fixar à parede arterial, o microrganismo gera inflamação aguda com infiltração neutrofílica, que leva à ativação de enzimas e ao enfraquecimento parietal^8^ e resulta em supuração e dilatação arterial^1^^,^^2^^,^^5^^,^^9^^,^^11^^,^^17^^,^^20^^,^^22^. O acometimento mais comum da aorta se deve ao vasa vasorum mais pronunciado em artérias mais calibrosas, o que favorece colonização bacteriana^8^^,^^11^^,^^14^^,^^19^.

Diagnóstico precoce é a chave para o sucesso terapêutico^6^^,^^8^^,^^9^^,^^12^^,^^16^. Classicamente, há febre^1^^,^^3^^,^^4^^,^^6^^-^8^,^^14^^,^^18^, dor abdominal/lombar e massa pulsátil^1^^,^^4^^,^^6^^-^8^,^^14^^,^^18^ na vigência de um quadro infeccioso (osteomielite, infecções do trato urinário, tuberculose, gastroenterite e infecções de partes moles)^1^^,^^4^^,^^6^^,^^11^^,^^14^ e quadros de imunossupressão por doenças ou medicamentos (câncer, insuficiência renal dialítica, HIV, diabetes, corticoides). Todavia, também existem casos assintomáticos^4^^,^^14^. Nos três casos relatados, houve dor abdominal; nos casos 1 e 2, havia antecedentes de quadro infeccioso, e no caso 1 também se identificou uso de medicamento imunossupressor. Exames laboratoriais geralmente mostram leucocitose^1^^,^^6^^,^^8^^,^^11^^,^^12^^,^^14^ e marcadores inflamatórios, como VHS e PCR, elevadas^6^^,^^12^, além de hemoculturas positivas^1^^,^^3^^,^^4^^,^^6^^,^^7^^,^^16^. Mesmo na fase aguda, a hemocultura pode ser negativa em até 50% dos casos^14^^,^^23^, principalmente se o paciente estiver recebendo antibióticos, o que é comum^8^^,^^14^^,^^15^. Na presente série, dois casos apresentaram hemoculturas positivas. Na angiotomografia, irregularidades parietais, dilatações saculares, alterações sugestivas de inflamação, massa/coleção líquida perivascular, formação de gás periaórtico, sinais de rotura livre ou contida e progressão rápida em exames seriados são frequentemente encontrados^1^^,^^4^^,^^5^^,^^8^^,^^9^^,^^11^^,^^12^^,^^14^^,^^17^^,^^24^. Na tomografia, abscessos hepáticos e esplênicos devem ser pesquisados^9^^,^^21^. É conveniente realizar ecocardiografia para investigar endocardite^4^. O exame tomografia por emissão de pósitrons com tomografia computadorizada (*positron emission tomography/computed tomography scan*, PET-CT SCAN) é uma ferramenta de alta acurácia diagnóstica, com grande sensibilidade, porém com especificidade prejudicada pelos resultados falso-positivos em casos de aneurismas inflamatórios e arterites^24^.

Não há consenso sobre como definir a etiologia infecciosa primária do aneurisma^5^^,^^15^^,^^17^. Sugere-se que o diagnóstico seja estabelecido pela combinação de quadro clínico, exames laboratoriais e achados tomográficos^1^^,^^6^^,^^12^^,^^17^^,^^21^ (Tabela 1). É fundamental esclarecer que o diagnóstico pode ser estabelecido na ausência de febre e hemoculturas positivas^1^^,^^3^^,^^8^^,^^9^^,^^15^^,^^16^^,^^23^. Após coleta de hemoculturas, iniciar antibioticoterapia para *Staphylococcus sp*, vancomicina, e antibioticoterapia para *Salmonella sp*: quinolonas ou cefalosporinas de terceira geração^1^^,^^4^^,^^11^^,^^12^^,^^15^^,^^16^^,^^23^, a ser modificada ou associada a antifúngicos, de acordo com os resultados. Não há consenso quanto à duração do tratamento^6^^-^8^,^^11^^,^^19^. A maioria dos autores sugere antibioticoterapia endovenosa pré-operatória por 1 a 6 semanas^4^^,^^6^^,^^12^. O tratamento clínico isolado, com antibióticos, resulta em mortalidade acima de 80%^13^^,^^14^^,^^23^.

**Tabela 1:** Critérios sugestivos de etiologia infecciosa do aneurisma.

**Apresentação clínica**	Dor abdominal/lombar
	Febre
	Sepse/choque
**Laboratório**	Proteína C reativa elevada
	Leucócitos elevados
	Cultura de sangue/tecido aórtico positiva
**Tomografia computadorizada**	Contorno sacular/multilobular
	Gás periaórtico
	Massa amorfa/coleção periaórtica
	Expansão rápida (dias)
	Ruptura
	Localização em segmento aórtico atípico (por exemplo, paravisceral)
	Aneurismas múltiplos

Fonte: compilado pelos autores, 2021.

O tratamento cirúrgico deve envolver abordagem por procedimento endovascular ou cirurgia convencional^1^^,^^3^^,^^6^^,^^7^^,^^9^^,^^14^^,^^15^^,^^21^^,^^23^. A terapia endovascular para aneurisma infeccioso da aorta torácica foi descrita em 1998^25^. Desde então, seguiram-se vários relatos^1^^,^^3^^,^^6^^,^^8^^,^^12^^,^^15^^,^^21^^,^^23^. Essa estratégia é menos invasiva, pois evitam-se dissecções em meio à anatomia comprometida pela infecção e não há pinçamento aórtico, o que em teoria beneficia pacientes com alto risco operatório^1^^,^^7^^,^^11^. Todavia, o implante da endoprótese em tecido infectado aumenta a incidência de complicações, entre as quais infecção da endoprótese^1^^,^^6^^,^^9^^,^^23^, seu mau posicionamento e consequentes *endoleaks* com potencial ruptura do aneurisma^4^^,^^8^. Outros desfechos indesejados incluem sepse, fístulas^8^ e crescimento do aneurisma^16^. É fato que o tratamento endovascular não permite a remoção do tecido infectado^9^^,^^12^^,^^16^^,^^23^ e tem grande risco de apenas postergar a cirurgia por via aberta, assim adicionando o explante da endoprótese a um ato operatório já de alta complexidade.

A cirurgia convencional, com morbimortalidade de até 44%^12^, é o padrão-ouro^11^^,^^12^^,^^16^^,^^22^, pois, embora proporcione recuperação mais lenta, apresenta menores taxas de reintervenção^3^^,^^6^. Além da ressecção aneurismática^9^^,^^12^^,^^13^, a cirurgia deve incluir extenso desbridamento retroperitoneal, ressecção aórtica circunferencial^9^^,^^13^^,^^16^^,^^22^^,^^23^ e revascularização *in situ* ou extra-anatômica com enxerto protético ou aloenxerto^1^^,^^9^^,^^11^^-^13^,^^16^^,^^22^. O *by-pass* extra-anatômico evita um enxerto em campo infectado; porém, pode ocorrer ruptura do coto aórtico, amputação do membro inferior ou reinfecção^8^^,^^13^. Já na revascularização *in situ*, feita com enxerto impregnado em prata ou antibióticos^13^, a anastomose em leito infectado implica risco de deiscência e formação de pseudoaneurisma^1^. Não há dados suficientes para comparar complicações entre enxertos *in situ* e extra-anatômicos, mas essas são mais comuns com o enxerto extra-anatômico^13^^,^^22^. Estudos mostram mortalidade de 5-49% para enxertos *in situ versus* 24-50% para extra-anatômicos; complicações infecciosas ocorrem em aproximadamente 20% dos casos em ambas as estratégias^11^.

A dissecção em meio a tecidos espessados/aderidos aumenta o risco de sangramento e de lesões iatrogênicas, principalmente da veia cava e ureteres. O uso de cateteres duplo J previamente pode ajudar a identificar os ureteres em meio ao retroperitônio espessado^21^^,^^22^, como feito no caso 1. Se a dissecção do colo distal não for possível, o clampeamento pode ser substituído pela oclusão endovascular das ilíacas com sondas de Foley^1^^,^^23^, como nos casos 1 e 3. Para isolar a prótese dos tecidos adjacentes, pode-se envolvê-la em pedículo vascularizado do grande omento^2^^,^^11^^,^^22^^,^^23^ como nos casos 1 e 3; a vascularização do omento também favorece a chegada do antibiótico à prótese^2^^,^^11^^,^^22^. Também são descritos o uso de aorta criopreservada de cadáver, indisponível no Brasil, e a reconstrução usando enxerto feito a partir de ambas as veias femorais^11^^,^^13^^,^^22^. Uso de cateteres duplo J para identificação dos ureteres, preparo de cólon com redução da necessidade de deslocar alças para fora da cavidade, acesso venoso central, monitorização invasiva da pressão arterial, uso de introdutor angiográfico 8F colocado na jugular interna para eventual infusão rápida de hemocomponentes, balanço hídrico positivo em pelo menos 1.000 mL antes da conclusão do ato operatório e aquecimento com manta térmica são alguns dos detalhes técnicos que melhoram os resultados cirúrgicos. Não há consenso a respeito da duração da antibioticoterapia pós-operatória^8^. Alguns autores recomendam 6 semanas^6^^,^^15^^,^^18^^,^^22^, enquanto outros sugerem 3 a 6 meses^4^^,^^6^^,^^7^^,^^11^^,^^12^^,^^15^, ou até antibioticoterapia vitalícia^6^^,^^12^^,^^22^.

Doenças raras e de tratamento complexo requerem troca de informação; a discussão com colegas que sejam referência em cirurgia aórtica e a troca de experiências em grupos que praticam inteligência coletiva^26^ é importante para aumentar as chances de êxito. Portanto, a etiologia infecciosa deve ser sempre considerada diante de febre e dor abdominal/lombar com massa pulsátil, principalmente na vigência de infecção comprovada ou imunossupressão por doenças/medicamentos e se houver elevação de leucócitos e de marcadores inflamatórios. Hemoculturas frequentemente são negativas. Imagens angiotomográficas sugestivas incluem dilatações saculares, coleções perivasculares e rotura contida. Esperar por quadros “clássicos” e hemoculturas positivas para iniciar o tratamento correto pode comprometer o prognóstico do paciente.

Para o controle pós-operatório, recomenda-se a realização de angiotomografias no primeiro e no sexto mês, e posteriormente, de forma anual, para avaliação de complicações e necessidade de reintervenção^12^.

Entre as limitações desta série, encontra-se o pequeno número de casos e a ausência de documentação dos exames de imagem realizados no seguimento pós-operatório dos pacientes.
